# A molecular subtyping associated with the cGAS-STING pathway provides novel perspectives on the treatment of ulcerative colitis

**DOI:** 10.1038/s41598-024-63695-4

**Published:** 2024-06-03

**Authors:** Chen Wang, Xin Gao, Yanchen Li, Chenyang Li, Zhimin Ma, Donglei Sun, Xiaonan Liang, Xiaolan Zhang

**Affiliations:** 1 Department of Gastroenterology, Hebei Key Laboratory of Gastroenterology, Hebei Institute of Gastroenterology, Hebei Clinical Research Center for Digestive Diseases, The Second Hospital of Hebei Medical University, Shijiazhuang, 050000 Hebei China; 2 Department of Respirology, The Second Hospital of Hebei Medical University, Shijiazhuang, 050000 Hebei China

**Keywords:** Ulcerative colitis, cGAS, STING, IFI16, Molecular subtype, Anti-TNF therapy, Biomarkers, Diseases, Gastroenterology

## Abstract

Ulcerative colitis (UC) is characterized by an abnormal immune response, and the pathogenesis lacks clear understanding. The cGAS-STING pathway is an innate immune signaling pathway that plays a significant role in various pathophysiological processes. However, the role of the cGAS-STING pathway in UC remains largely unclear. In this study, we obtained transcriptome sequencing data from multiple publicly available databases. cGAS-STING related genes were obtained through literature search, and differentially expressed genes (DEGs) were analyzed using R package limma. Hub genes were identified through protein–protein interaction (PPI) network analysis and module construction. The ConsensuClusterPlus package was utilized to identify molecular subtypes based on hub genes. The therapeutic response, immune microenvironment, and biological pathways of subtypes were further investigated. A total of 18 DEGs were found in UC patients. We further identified *IFI16*, *MB21D1 (CGAS)*, *TMEM173 (STING)* and *TBK1* as the hub genes. These genes are highly expressed in UC. *IFI16* exhibited the highest diagnostic value and predictive value for response to anti-TNF therapy. The expression level of IFI16 was higher in non-responders to anti-TNF therapy. Furthermore, a cluster analysis based on genes related to the cGAS-STING pathway revealed that patients with higher gene expression exhibited elevated immune burden and inflammation levels. This study is a pioneering analysis of cGAS-STING pathway-related genes in UC. These findings provide new insights for the diagnosis of UC and the prediction of therapeutic response.

## Introduction

Ulcerative colitis (UC) is a chronic inflammatory disease that can affect the rectum and extend to pancolitis, significantly impacting the quality of life. The global prevalence of UC is expected to reach 5 million cases by 2023, with an increasing incidence rate each year^1^. While the development of biologics targeting tumour necrosis factor (TNF) has provided hope for UC patients, the rates of primary non-response and secondary loss of response remain high^2^. Other emerging biological therapies and small molecule drugs, such as vedolizumab and Janus kinase inhibitors, have shown limited therapeutic efficacy, with clinical remission rates ranging from only 10–30%^3,4^. Long-term treatment with these drugs may have side effects such as severe infections, neurological disorders, malignancies and thrombosis. Therefore, revealing the pathogenesis and exploring new therapeutic targets are crucial for patients with UC.

The pathogenesis of UC is not fully understood and may be influenced by a combination of genetic susceptibility, environmental factors, gut microbiota, and immune response^5^. As the first defense of host immunity, innate immunity acts first when pathogens invade the body^6^, and plays an important role in the development of UC. Recent studies found that Cyclic GMP-AMP (cGAMP) synthase (cGAS)-stimulator of interferon genes (STING) signaling pathway is one of the important pattern recognition and effector pathways in the innate immune system. Cyclic GMP-AMP (cGAMP) synthase (cGAS) is activated when it recognizes and binds to double-stranded DNA (dsDNA), resulting in the synthesis of 2´3´-cGAMP. 2ʹ3ʹ-cGAMP as a second messenger binds and activates stimulator of interferon genes (STING), which subsequently triggers a signalling cascade to activate the innate immune system through production of immune and inflammatory factors, such as type I interferon (IFN-I)^7,8^. Abnormal cGAS-STING pathway activation may lead to the occurrence of autoimmune diseases. Increasing evidence suggests that this pathway plays a significant role in inflammation-related diseases^9,10^. Consequently, the cGAS-STING signaling pathway is expected to be a new target for the treatment of autoimmune diseases.

Inhibition of cGAS-STING pathway to reduce abnormal inflammatory response has become a new research direction for the treatment of various autoimmune diseases such as systemic lupus erythematosus^11^. The latest study shows that the clinical development of inhibitor has begun, and clinical trials are planned to begin^12^. Interestingly, previous study has reported that the cGAS-STING pathway is involved in the pathogenesis, progression, and therapeutic response of UC^13^. The level of STING is increased in the colon of patients with UC^14^. However, our understanding of the role of the cGAS-STING pathway in UC process is remains limited. This lack of knowledge may be a key factor affecting future clinical diagnosis and treatment.

In this study, we aimed to systematically summarized the cGAS-STING pathway-related genes and identified hub genes in UC. In addition, patients with UC were classified based on the cGAS-STING pathway-related genes. The correlations between hub genes and anti-TNF therapy efficacy were further investigated and validated. Finally, we analyzed the expression of hub genes in cancer.

## Materials and methods

### Patients

Human colon tissue samples were obtained from patients with UC prior to the treatment of infliximab. The patients with UC were divided into two groups based on their response to infliximab. The participants were recruited from the Second Hospital of Hebei Medical University. All procedures were conducted in accordance with the principles of the Declaration of Helsinki and approved by the Ethics Committee of the Second Hospital of Hebei Medical University.

### Data collection and processing

The microarray data and clinical information of the patients were obtained from multiple public databases. All databases are listed in Table 1. We used ulcerative colitis as keyword to retrieve gene expression profiles and corresponding clinical data from the Gene Expression Omnibus (GEO) database (http://www.ncbi.nlm.nih.gov/geo). The GSE38713 dataset contains RNA sequencing results of colonic mucosal tissues from 15 UC patients with active disease and 13 healthy controls^15^. The GSE47908 dataset contains RNA sequencing results of colonic mucosal tissues from 39 patients with UC and 15 controls^16^. After normalizing the data, the batch effect was removed using ComBat function from the "sva" package between the two datasets^17^. The "limma" package (Version 3.42.2) was used to identify differentially expressed genes (DEGs) between inflammatory and healthy tissues^18^. The adjust *P* value < 0.05 were statistically signifcant.

**Table 1:** The information of all datasets.

Dataset	Platform	Sample and disease	Anti-TNF therapy	Details
GSE47908	GPL570	Active UC vs Controls	No	–
GSE38713	GPL570	Active UC vs Controls	No	–
GSE107499	GPL15207	Lesional tissue vs Non-lesional tissue	No	–
GSE16879	GPL570	Responder vs Non-responder	Yes	Treatment process available
GSE12251	GPL570	Responder vs Non-responder	Yes	–
GSE73661	GPL6244	Responder vs Non-responder	Yes	Treatment process available
GSE31106	GPL1261	Mice from colitis to cancer	No	–
GSE3629	GPL570	UC vs UC-ca	No	–
XENA	–	CRC vs Normal	No	TCGA_GTEx-READ
K–M plotter	–	Pan-cancer	No	Immunotherapy data

### Identification of cGAS-STING pathway-related differentially expressed genes

A literature search identified 30 genes that have been implicated in the cGAS-STING pathway (Supplementary Table S1). To demonstrate the reliability of the data, we used the original gene ID from the GEO data. For instance, CGAS and STING were previously known as MB21D1, TMEM173. Next, we used a Venn diagram tool to determine the overlap between the DEGs and the 30 genes related to the cGAS-STING pathway.

### Protein–protein interaction network construction and evaluation of hub genes

Using a protein–protein interaction network (PPI network, Version 11.5, https://string-db.org) to study interactions between DEGs, we defined statistical significance as an interaction score > 0.7 and obscured individual target protein nodes. Subsequently, the identification of hub genes was performed using the molecular complex detection (MCODE) plug-in in Cytoscape software (Version 3.9.1). To assess the diagnostic value of hub genes, we employed receiver operating characteristic curves (ROC) and the area under the curve (AUC) by "pROC" package.

### The relationship between hub genes and anti-TNF therapy

GSE16879 and GSE12251 were merged to investigate the correlation between anti -TNF therapy and hub genes expression^19,20^. GSE73661 was used as the validation database^21^. Cluster analysis was performed using the "ConsensusClusterPlus" package, and DEGs between the various clusters were found using the "limma" package (adjusted *P* < 0.05).

### Functional enrichment analysis

The "clusterProfiler" package was used to perform Gene Ontology (GO), Kyoto Encyclopedia of Genes and Genomes (KEGG), and Gene Set Enrichment Analysis (GSEA) based on the DEGs between various clusters. The "Hallmarks" pathway was chosen for GSEA.

### Immune landscape analysis

We used single sample gene set enrichment analysis (ssGSEA) through "GSVA" package^22^ to evaluate the difference of immune cell infiltration and alternations of immune-related pathways between different clusters.

### Kaplan–Meier (KM) plotter database

The Kaplan Meier plotter (http://kmplot.com/analysis) online database was used to analysis the relationship between IFI16 and immunotherapy outcomes. The hazard ratio (HR) with 95% confidence intervals and log rank *P* value were calculated.

### Immunohistochemistry staining

Colon tissue samples were fixed in 4% paraformaldehyde and subsequently embedded in paraffin. The tissue was sliced into 4 μm thickness for immunostaining. Sections were used for analyzing the expression of IFI16 with IFI16 Rabbit mAb (1:400 dilution; 14970, Cell Signaling Technology, MA, USA). Images were captured by CX40 optical microscope (Olympus, Tokyo, Japan) and and three fields were randomly selected for analysis. The expression of IFI16 in each of the randomly selected fields was quantified using ImageJ.

### Statistical analysis

The statistical analyzes were performed in R software (version 3.6.0) and GraphPad Prism (version 9.0.0). Use the “ggplot2” package (Version 3.3.3) to visualize the data, and choose the appropriate statistical methods based on the features of the data format for statistical analysis (“stats” package and “car” package). Wilcoxon rank-sum test or T-test was used to analyze the continuous variables according to the normality. Fisher's exact test was used to examine the differences of categorical variables. *P* < 0.05 was considered statistically significant.

### Ethics statement

The studies involving human participants were reviewed and approved by Ethics Committee of the Second Hospital of Hebei Medical University. The patients provided their written informed consent to participate in this study.

## Results

### Identification of cGAS-STING pathway expression landscape in patients with UC

**Figure 1:**
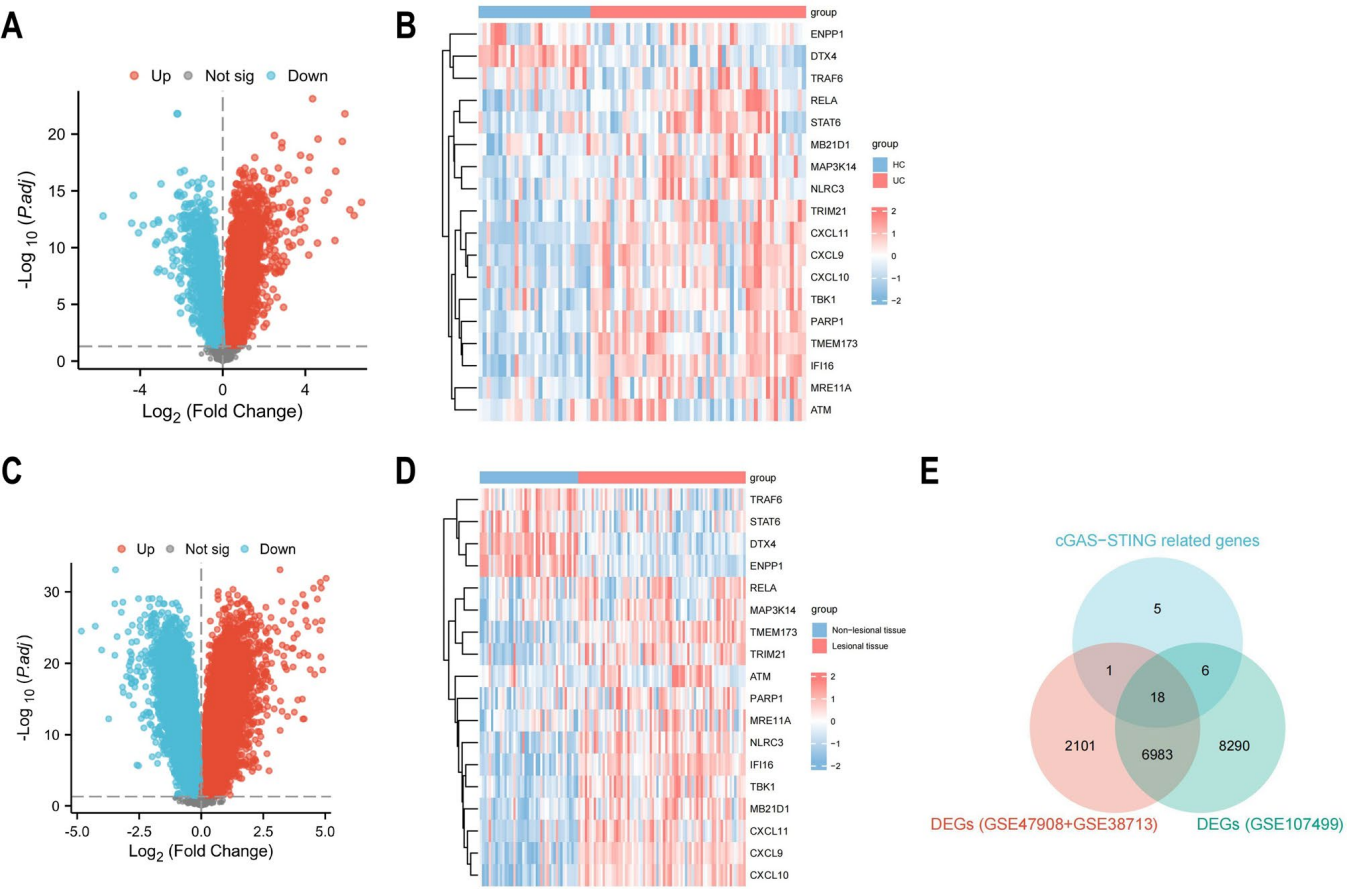
Identification of cGAS-STING pathway expression landscape. (**A**–**D**) The volcanic map and heat map show differential gene expression of GSE47908 + GSE38713 (**A**, **B**) and GSE107499 (**C**, **D**). (**E**) Venn diagrams shows intersected DEGs.

To compare mRNA expression differences between UC patients and controls, datasets GSE38713 and GSE47908 were combined. Batch effect elimination was performed on the merged dataset before data analysis (Supplementary Fig. S1). In a comparative analysis of mucosal gene expression between UC patients and controls, the volcano map and heat map respectively display the cGAS-STING pathway expression landscape (Fig. 1A–D). A total of 18 genes were selected for further investigation based on the overlapping genes found in all three circumstances (Fig. 1E). In summary, patients with UC showed decreased mRNA expression of *DTX4, ENPP1, TRAF6*, and increased mRNA expression of *TMEM173 (STING), IFI16, CXCL11, CXCL9, PARP1, RELA, CXCL10, TBK1, TRIM21, MB21D1 (CGAS), MAP3K14, NLRC3, MRE11A, ATM*.

### Identification of hub genes and diagnositc value

**Figure 2:**
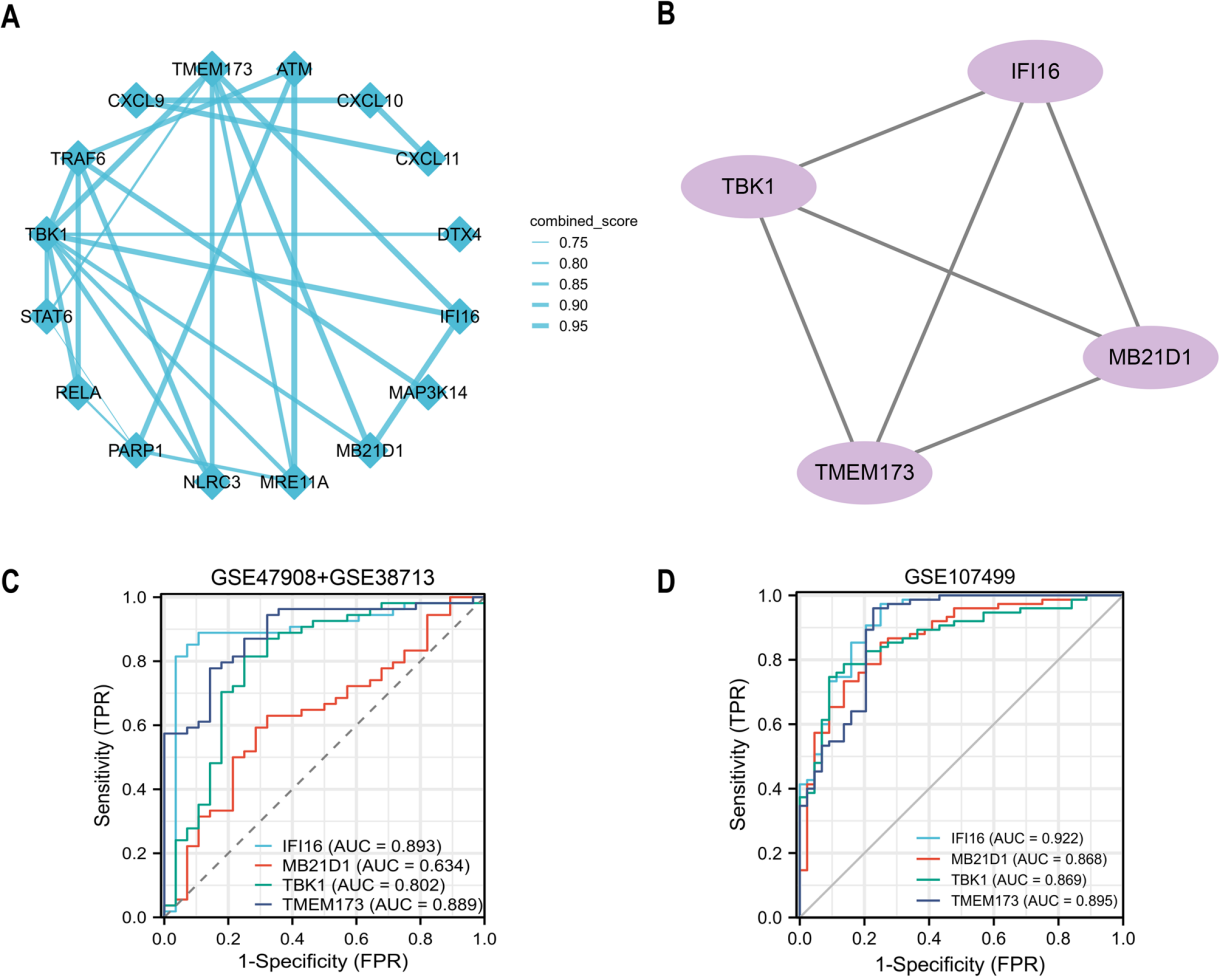
Identification of cGAS-STING pathway-related hub genes in UC. (**A**) PPI network of 18 overlapping DEGs. (**B**) 4 hub genes are identified based on Cytoscape plug-in Mcode. (**C**, **D**) ROC curve show the diagnostic values of hub genes in UC.

We established interactive associations between 18 DEGs using the PPI network (Fig. 2A). By using Cytoscape's MCODE plug-in, we found a module made up of four hub genes. *IFI16*, *MB21D1 (CGAS)*, *TMEM173 (STING)*, and *TBK1* were identified as hub genes that are up-regulated in inflammation (Fig. 2B). The diagnostic value of four hub genes for UC was next assessed, and we discovered that *IFI16* has the highest value (AUCs of 0.893 and 0.922, respectively) (Fig. 2C,D).

### Predictive values and expression changes of hub genes in anti-TNF treatment

**Figure 3:**
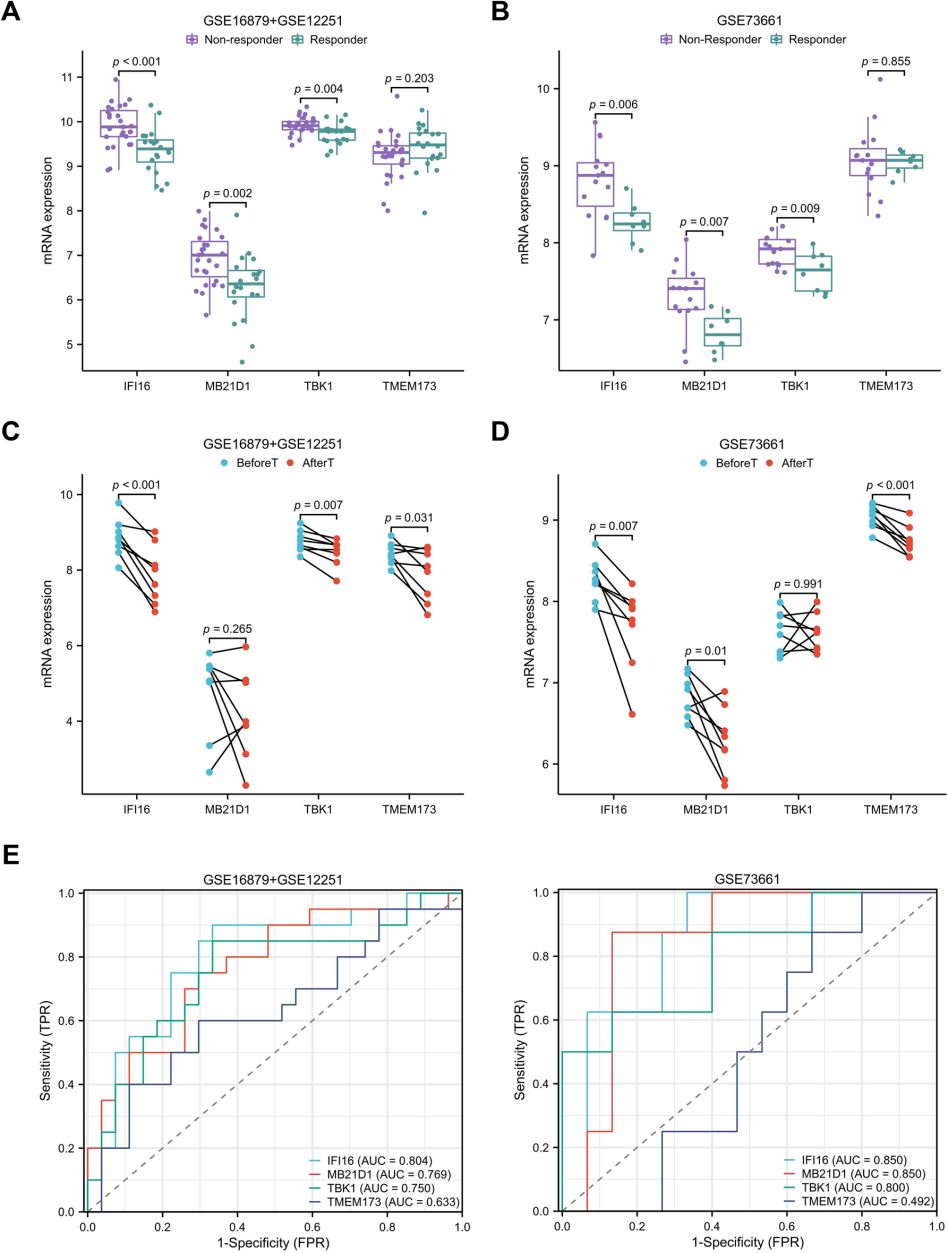
Expression differences of hub genes between responders and non-responders with anti-TNF treatment. (**A**, **B**) Comparison of hub gene expression between responders and non-responders with anti-TNF therapy based on GSE16879 + GSE12251 datasets and GSE73661. (**C**, **D**) Comparison of hub gene expression in responders before and after treatment. (**E**) ROC curve show the predictive values of hub genes in the anti-TNF treatment.

Three datasets (GSE16879, GSE12251, and GSE73661) provided the data for UC patients who had received the anti-TNF therapy. To look into the relationship between hub genes expression and anti-TNF therapy, GSE16879 and GSE12251 were combined. We examined the expression of hub genes in responders and non-responders at the start of anti-TNF treatment. It was discovered that non-responders expressed *IFI16*, *MB21D1 (CGAS),* and *TBK1* at higher levels than responders (Fig. 3A,B). Anti-TNF therapy was found to decrease the expressions of *IFI16*, and *TMEM173 (STING)* in the majority of responders (Fig. 3C,D). Meanwhile, *IFI16* has the highest predictive value, the AUC of *IFI16* reached 0.804 and 0.850 (Fig. 3E). The results suggest that poor anti-TNF therapy outcomes are associated with a high level of the cGAS-STING pathway, and *IFI16* is a promising predictive biomarker for this clinical therapeutic decision.

### Expression of IFI16 in UC patients receiving infliximab therapy

**Figure 4:**
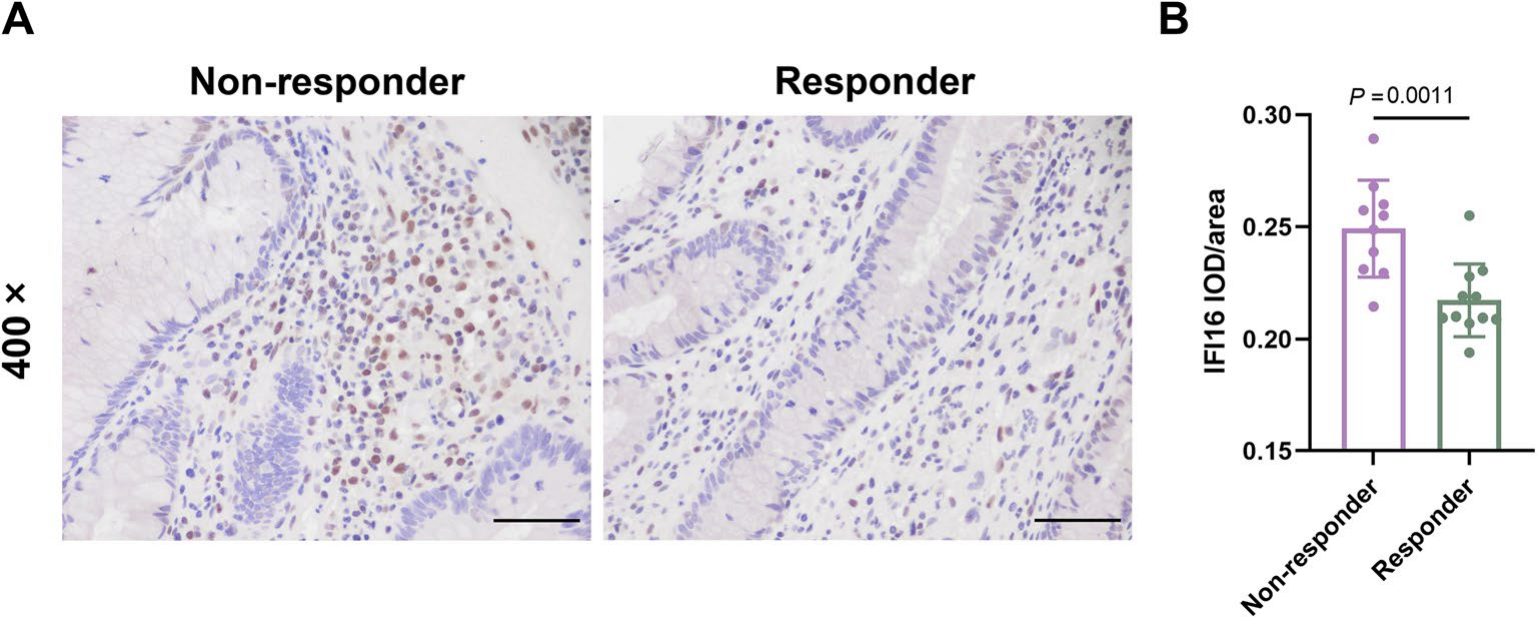
IFI16 expression in UC patients treated with infliximab. (**A**) Representative images of IFI16 immunohistochemistry staining (scale bar = 50 µm). (**B**) Expression of IFI16 in colon tissues. Data are presented as mean ± SD.

We collected colon tissue samples from 21 patients with UC to investigate the changes in IFI16 expression between infliximab responders and non-responders. The baseline characteristics of responders and non-responders are shown in Table 2. The results of the immunohistochemistry staining showed that non-responders had higher levels of IFI16 expression compared to responders (Fig. 4A,B).

**Table 2:** Base-line characteristics of UC patients.

Characteristics	Responder (n = 11)	Non-responder (n = 10)	*P* value
Age (years, mean ± sd)	39.82 ± 16.22	35.10 ± 11.14	0.451
Gender (n, M/F)	9/2	6/4	0.362
BMI (mean ± sd)	21.16 ± 4.01	20.97 ± 3.60	0.909
ESR [mm/h, median (IQR)]	26.00 (3.00, 36.00)	37.00 (15.50, 87.50)	0.179
CRP [mg/L, median (IQR)]	12.20 (3.00, 61.50)	13.40 (3.00, 35.05)	0.856
Hb [g/L, mean ± sd]	123.4 ± 29.57	103.1 ± 30.82	0.141
PLT [10^9^/L, median (IQR)]	242.0 (193.0, 363.0)	228.5 (152.3, 388.5)	0.557
ALB [g/L, mean ± sd]	34.89 ± 8.089	29.82 ± 5.997	0.122
FC [μg/g, median (IQR)]	200.0 (79.70, 252.9)	195.9 (4.00, 465.2)	0.742
UCEIS [median (IQR)]	5 (2, 7)	5 (1, 8)	0.847

### Identification of different subtypes based on hub genes

**Figure 5:**
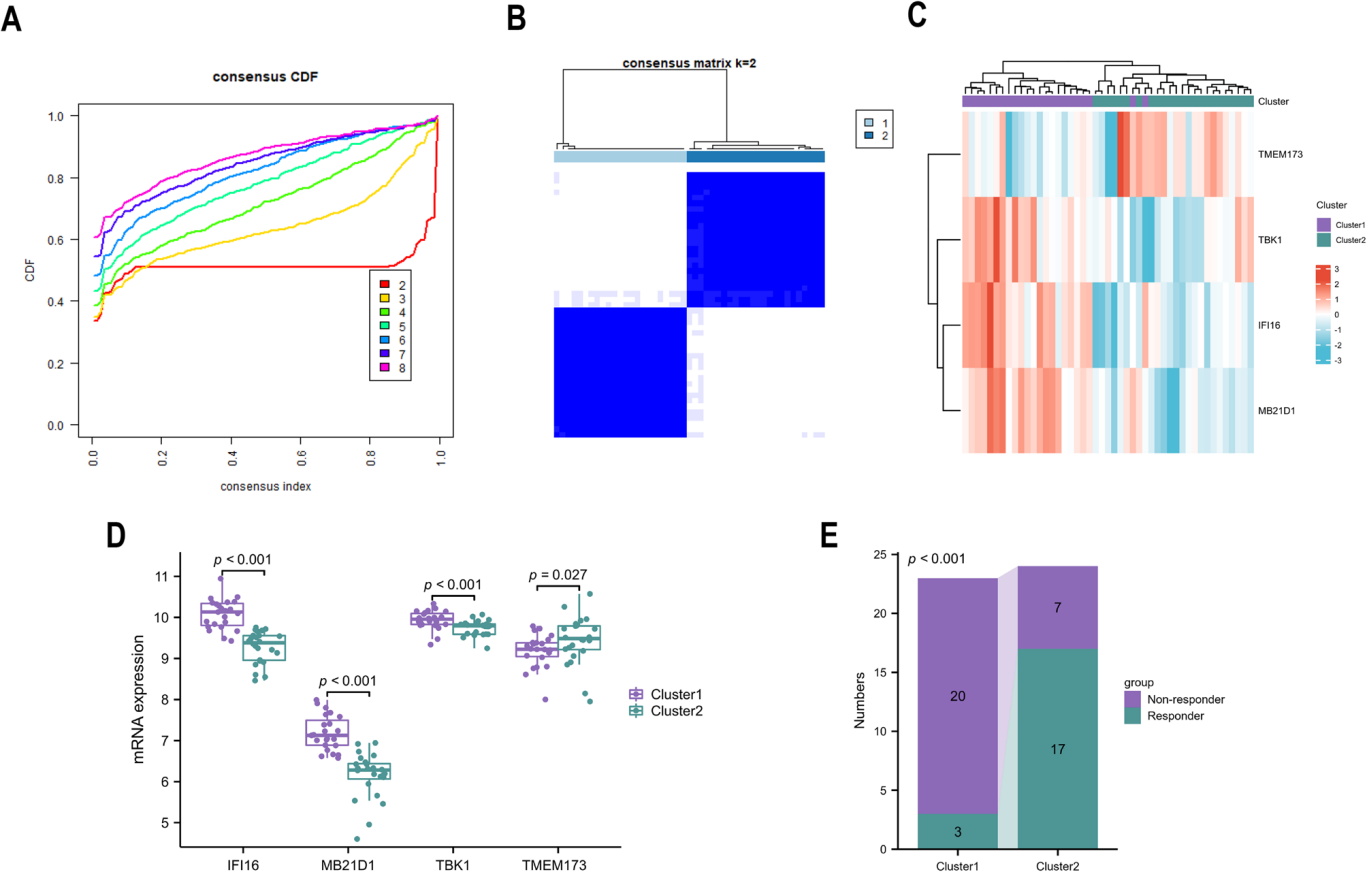
Classification of UC patients according to hub genes. (**A**) Using unsupervised consensus clustering, patients with UC of GSE16879 + GSE12251 are divided into two groups based on hub genes. (**B**) The item-consensus plot reveals that K = 2 is the best grouping. The expression of hub genes in each cluster is depicted by a heatmap (**C**) and box plots (**D**). (**E**) The stacked column chart shows the proportion of respondents and non-responders in two groups.

A cluster analysis of UC patients undergoing anti-TNF therapy was conducted based on four hub genes. The best clusters were observed when K = 2 (Fig. 5A,B). While *TMEM173 (STING)* was downregulated in cluster 1, *IFI16*, *MB21D1 (CGAS)*, and *TBK1* were generally upregulated in cluster 1 (Fig. 5C,D). Compared to patients in cluster 1, patients in cluster 2 exhibit a greater response rate to anti-TNF treatment (Fig. 5E).

### Functional enrichment and immunological infiltration analysis

**Figure 6:**
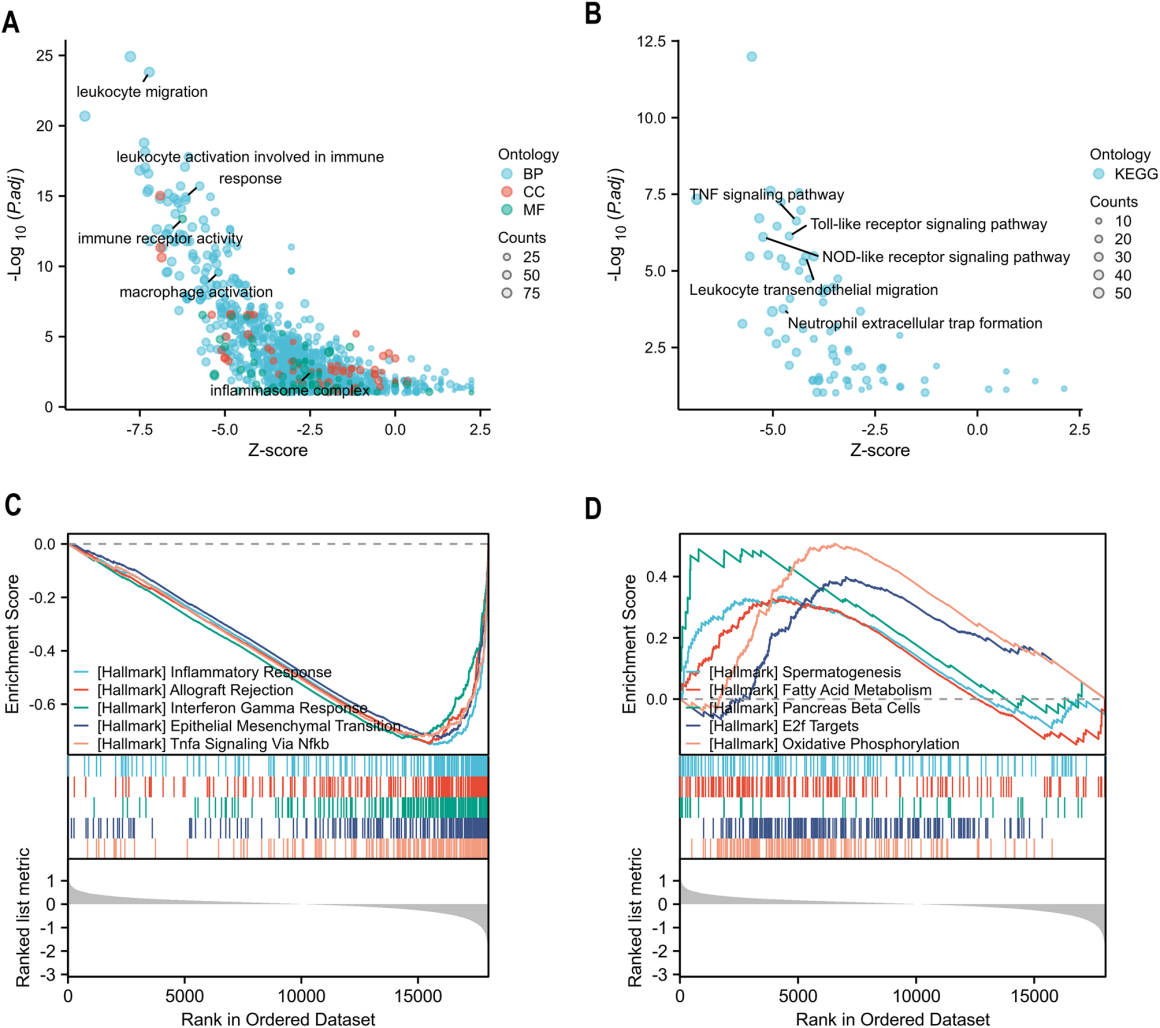
Pathway enrichment analysis in two clusters. (**A**) The GO analysis's enriched pathways (BP stands for biological process, MF for molecular function, and CC for cellular components). (**B**) KEGG analysis. (**C**) Enriched pathways according to GSEA analysis.

Additional functional enrichment analysis was performed in order to clarify the variations in function between the two clusters (Supplementary Table S2). Multiple immune-related pathways were shown to be enhanced in cluster 1 by GO analysis, including leukocyte migration, leukocyte activation involved in immune response, immune receptor activity, macrophage activation, and inflammsome complex (Fig. 6A). Based on KEGG pathway analysis, TNF signaling pathway, Toll-like receptor signaling pathway, and NOD-like receptor signaling pathway were all active in cluster 1 (Fig. 6B). According to the GSEA results, cluster 1 had increased levels of INFLAMMATORY_RESPONSE, ALLOGRAFT REJECTION, INTERFERON_GAMMA_RESPONSE, EPITHELIAL MESENCHYMAL TRANSITION and TNFA_SIGNALING_VIA_NFKB, while cluster 2 had increased levels of SPERMATOGENESIS, FATTY_ACID_METABOLISM, PANCREAS BETA CELLS, E2F TARGETS and OXIDATIVE_PHOSPHORYLATION (Fig. 6C,D).

**Figure 7:**
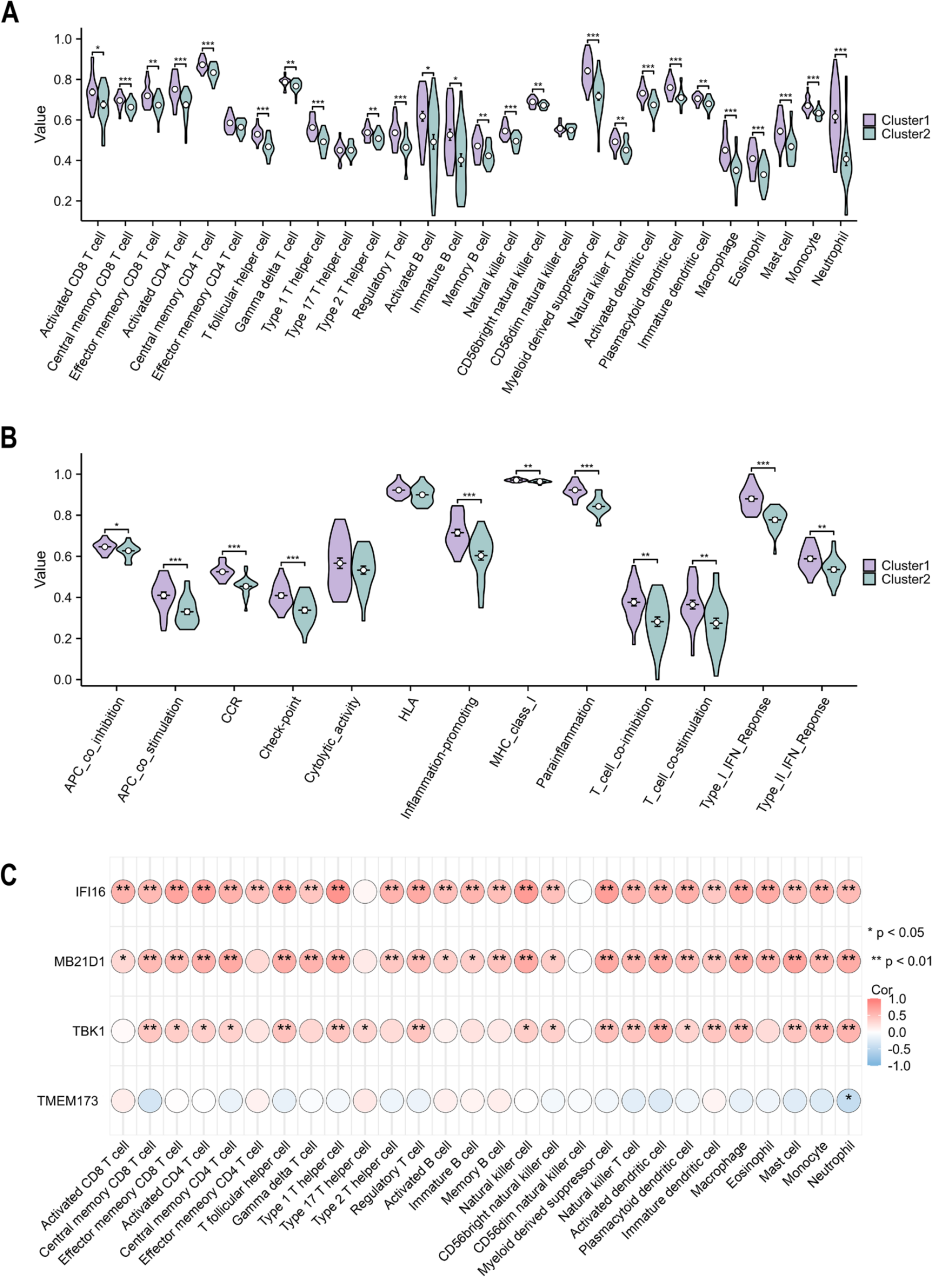
Comparison of immune microenvironment between 2 clusters. (**A**) Immune cell infiltration analysis. (**B**) Immune reaction. (**C**) The relationship between hub gene expression and immune infiltration.

Immune infiltration analysis showed similar results. Using ssGSEA, the abundance of 28 immune cells was calculated. Cluster 1 was concentrated in both innate and adaptive immune cells (Fig. 7A). Similarly, cluster 1 was enriched in several immune-related pathways, including those that promote inflammation and the IFN response (Fig. 7B). After examining the connection between immune infiltration and hub gene expression, we discovered that *IFI16* is linked to the greatest level of immune cell infiltration (Fig. 7C). According to these investigations, cluster 1 exhibited a higher immunological burden and degree of inflammation than cluster 2.

## cGAS-STING may inhibit *cancer* development by enhancing immune infiltration

**Figure 8:**
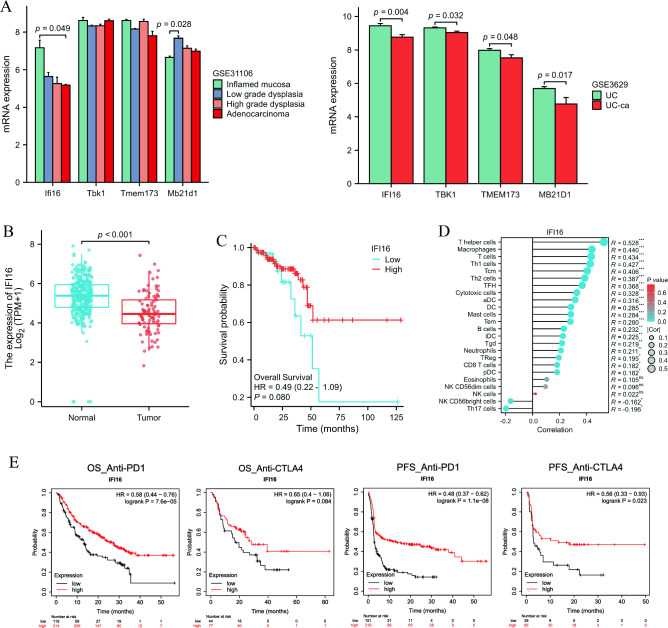
The role of cGAS-STING pathway-related hub genes in cancer. (**A**) Expression changes of hub genes during colitis-carcinoma transformation in mice and humans. (**B**) Expression of IFI16 in tumor tissues from the rectal cancer database. (**C**) Survival curves for overall survival. (**D**) Immune cell infiltration analysis. (**E**) The K–M survival curve shows that patients received immunotherapy with high expression of IFI16 are associated with better prognosis.

The development of colitis-associated colorectal cancer (CAC) is one of the most serious outcomes of UC. We investigated the expression of hub genes associated with the inflammation-carcinogenesis process. Interestingly, we found that the expression of the cGAS-STING pathway actually decreased during the process of colitis-carcinoma transformation. Particularly, the gene expression of IFI16 (murine homolog IFI204) was significantly decreased (Fig. 8A). This phenomenon also occurs in sporadic rectal cancer (Fig. 8B). The Kaplan–Meier survival curve demonstrates that patients with high expression of IFI16 have better overall survival (Fig. 8C). Additionally, immune infiltration analysis reveals that IFI16 is associated with more abundant immune infiltration (Fig. 8D). It is widely recognized that hot tumors with abundant immune infiltration are crucial for the effectiveness of immunotherapy. Therefore, we hypothesize that IFI16 could serve as a biomarker for the application of immunotherapy. To explore this further, we examined the relationship between IFI16 and immunotherapy outcomes using the KM plotter database. The results indicate that patients with high IFI16 expression who received PD-1 and CTLA4 monoclonal antibodies had improved overall survival and progression-free survival (Fig. 8E). These findings suggest that IFI16 may play a role in regulating tumor progression through the immune microenvironment.

## Discussion

The cGAS-STING pathway is crucial for mammalian cells to detect exogenous DNA and activate innate immune responses. This pathway is widely associated with various pathophysiology processes such as infections, stress, and inflammation^23^. UC is a result of genetic susceptibility, environmental factors, and gut microbiota interactions, leading to a weakening of the intestinal barrier function and activation of the intestinal immune response^24^. It has been found that cGAS-STING pathway is correlated with the development of UC^14,25,26^. Therefore, this study explored the role of cGAS-STING pathway-related genes in the process of UC. Furthermore, we also discovered that these genes play a significant role in predicting drug efficacy and the progression of colitis-carcinoma transformation.

Firstly, we searched for 30 genes related to the cGAS-STING pathway by retrieving relevant papers. Meanwhile, we screened the RNA sequencing data of UC from the GEO database. From the sequencing data, we identified 18 DEGs that overlapped with the cGAS-STING pathway-related genes. Based on these genes, we comprehensively mapped the cGAS-STING pathway in UC, suggesting that these genes may play important roles in the development of UC. Mechanistically, cGAS is a primary sensor of cytosolic DNA. Upon binding dsDNA, cGAS dimers assemble on dsDNA, resulting in enzymatic activation of cGAS and synthesis of cGAMP. cGAMP binds to STING and activates tank-binding kinase 1 (TBK1), promoting the autophosphorylation of TBK1, phosphorylation of STING at Ser366, and recruitment of interferon regulatory factor 3 (IRF3), then induce the release of IFN-I, NF-κB mediated proinflammatory cytokines and chemokines^8,9^. DTX4 is a negative regulator of TBK1. Previous studies have shown that NLRP4 recruited the E3 ubiquitin ligase DTX4 to TBK1 for Lys48 (K48)-linked Lys670 polyubiquitination, which led to the degradation of TBK1^27^. Additionally, ENPP1 regulates extracellular cGAMP to reduce STING signaling^28^. Consistent with our study, these genes may be involved in cGAS-STING signaling pathways as negative regulators in UC. However, the expression of STAT6 varied in different databases. It has been reported that activation of the cGAS-STING pathway recruits STAT6 to the endoplasmic reticulum, resulting in STAT6 phosphorylation by TBK1 and Tyr(641) at Ser(407) and subsequent transfer to the nucleus to mediate immune signaling^29^. STAT6 activation has been detected in the inflamed colonic epithelium of patients with active inflammatory bowel disease (IBD). By weakening the function of intestinal epithelial barrier, non-hematopoietic STAT6 increases intestinal permeability, aggravates experimental colitis induced by dextran sulphate sodium (DSS) and further leads to CAC^30^. In conclusion, our study provides a comprehensive summary and identification of genes associated with the cGAS-STING pathway in UC. However, the role of these genes in UC has not been fully revealed, and further research in the future holds significant clinical value.

Next we identified four hub genes associated with the cGAS-STING pathway in UC, including *IFI16*, *MB21D1 (CGAS)*, *TMEM173 (STING)*, and *TBK1*. *MB21D1 (CGAS)*, *TMEM173 (STING)*, and *TBK1* are considered to be the key signaling molecules in cGAS-STING pathway^31^. Recent studies have shown that the cGAS-STING pathway is activated in patients with UC^32^. Animal experiments found that dysbiosis can promote the accumulation of STING in intestinal myeloid cells, resulting in intestinal inflammation^14^. These results suggested that the cGAS-STING pathway may play an important role in UC. In addition, IFI16 (interferon-γ inducible protein 16) belongs to the PYHIN-200 family, which encodes evolutionarily related human proteins^33^. The murine homolog of IFI16 is IFI204 (p204)^34^. While there have been numerous studies on the function of cGAS in recognizing exogenous DNA, fewer studies have focused on the function of IFI16 in the cGAS-STING-TBK1 pathway. It has been found that IFI16 and cGAS work synergistically to promote STING activation. Moreover, IFI16 is essential for cGAMP-stimulated downstream signaling, which promotes TBK1 recruitment and activation in the STING complex^35,36^. Given the unclear pathogenesis and complexity of UC, diagnosing the disease remains challenging. Therefore, convenient and economical diagnostic methods are urgently needed. In our analysis, we found that IFI16 had the highest diagnostic efficacy among the four hub genes for UC. Therefore, IFI16 is expected to be a biomarker for the diagnosis of UC. Additionally, IFI16 has the potential to serve as a diagnostic and prognostic biomarker for various diseases, including tumors and rheumatoid arthritis^37,38^.

Early identification of non-responders in UC treatment remains a challenge that needs to be addressed. Our study focused on examining the expression of hub genes in patients who responded or did not respond to anti-TNF therapy. We found that IFI16 exhibited the highest predictive value in determining whether a patient would respond to the treatment or not. Furthermore, we observed a decrease in the expression of genes in responders during treatment, which could be attributed to the inhibition of the cGAS-STING pathway by anti-TNF drugs. Notably, a clinical study found that Anti-IFI16 IgG titers may be of great clinical relevance. Patients with low Anti-IFI16 IgG titers prior to infliximab treatment demonstrated a significantly higher probability of clinical response or remission^39^. Subsequently, we validated this finding using human samples and discovered that non-responders had higher levels of IFI16 protein compared to responders. These findings suggest that IFI16 has the potential to serve as a promising biomarker in the future.

Different sets of functional genes can be used to group samples, with each cluster having distinct pathogenic mechanisms and clinical prognostic features^40,41^. Through unsupervised consensus clustering, we have identified two subtypes based on cGAS-STING pathway-associated genes. Cluster1, which has a high proportion of non-responders to anti-TNF therapy, exhibits abundant immune cell infiltration, activated immune responses, and inflammatory responses. These findings indicate that cGAS-STING pathway-related genes are involved in immune regulation in UC.

CAC is the most serious complications of UC. The process of CAC typically involves inflammation, dysplasia, and carcinoma. Inflammation is the initiator of CAC, while abnormal immune response is the trigger of inflammation-carcinogenesis transformation^42,43^. Encouragingly, the incidence of CAC appears to be decreasing over time, which could potentially indicate advancements in medical treatment and colonoscopy^44–46^. However, it is important to note that CAC still remains the primary cause of death and colectomy in patients with UC^42^. Interestingly, the cGAS-STING signaling pathway may have different roles in inflammation and cancer. On the one hand, activation of cGAS-STING signaling is associated with the severity of intestinal inflammation. On the other hand, it plays a key role in preventing tumorigenesis and infection^13^. Colorectal cancer (CRC) patients with high STING expression had increased intratumoral CD8^+^ T cells infiltration and decreased frequency of lymphovascular infiltration. Additionally, patients with higher STING expression had longer overall survival and recurrence-free survival compared to those with lower STING expression^47^. Another study found that STING^−/−^ mice alleviated colitis but exacerbated the development of CAC. STING inhibited the development of CAC by regulating tumor cell proliferation, adhesion, invasion and inflammatory responses^48^. This is consistent with our finding that IFI16 expression decreases in inflammation-carcinogenesis transformation process. Furthermore, tumor patients with high IFI16 expression had better overall survival and immunotherapy outcomes. In conclusion, we found that cGAS-STING pathway-related genes may play different roles in colitis and tumors. IFI16 is expected to be a biomarker for predicting the efficacy of immunotherapy in CRC patients.

The mechanism of cGAS-STING pathway in UC has not been fully elucidated. In this study, we investigated the role and function of cGAS-STING pathway in UC at the gene level. We also demonstrated its potential therapeutic predictive value and provided a new perspective for further research. However, our study has some limitations. Although we observed that cGAS-STING pathway-related genes might have different roles in UC and CAC, we did not further explore the underlying reasons in depth. Our study is mainly based on online database, and we will probably further clarify the roles of cGAS-STING pathway genes in UC and CAC by cell and animal experiments in the future.

## Conclusion

In conclusion, our study systematically summarized the cGAS-STING pathway-related genes in UC and identified *IFI16*, *MB21D1 (CGAS)*, *TMEM173 (STING)*, and *TBK1* as hub genes. These hub genes are associated with the immune landscape, subtypes of UC patients and the effects of drug treatment. Notably, we identified *IFI16* as a promising biomarker for predicting the efficacy of anti-TNF therapy and might playing an important role in CAC. Our findings provide new insights for exploring the molecular mechanisms and therapy of UC and cancer. Further exploration and validation of mechanisms will be necessary.

### Supplementary Information

Supplementary Legends.

Supplementary Figure S1.

Supplementary Table S1.

Supplementary Table S2.

## Data Availability

The datasets generated during the current study are available from the corresponding author on reasonable request.

## Abbreviations

UC: Ulcerative colitis
TNF: Tumour necrosis factor
IFN-I: Type I interferon
cGAS: Cyclic GMP-AMP synthase
STING: Stimulator of interferon genes
GEO: Gene expression omnibus
DEGs: Differentially expressed genes
MCODE: Molecular complex detection
PPI: Protein–protein interaction
ROC: Receiver operating characteristic curves
AUC: Area under the curve
GO: Gene ontology
KEGG: Kyoto encyclopedia of genes and genomes
GSEA: Gene set enrichment analysis
ssGSEA: Single sample gene set enrichment analysis
HR: Hazard ratio
CAC: Colitis-associated colorectal cancer
TBK1: Tank-binding kinase 1
IRF3: Interferon regulatory factor 3
IBD: Inflammatory bowel disease
DSS: Dextran sulphate sodium
IFI16: Interferon-γ inducible protein 16
CRC: Colorectal cancer
