# Supplementary material for: A molecular subtyping associated with the cGAS-STING pathway provides novel perspectives on the treatment of ulcerative colitis
Source: Sci Rep. 2024 Jun 3;14:12683. doi: 10.1038/s41598-024-63695-4 (PMC11148070; doi:10.1038/s41598-024-63695-4)
Supplement: Supplementary file 1 — Supplementary Legends. [file 41598_2024_63695_MOESM1_ESM.docx]

**Supplementary Fig. 1 GEO dataset preprocessing. (A)** Gene expression level of the dataset before batch effect correction. **(B)** Gene expression level of the dataset after batch effect correction.

**Supplementary Table S1 Genes associated with cGAS-STING pathway.**

**Supplementary Table S2 Pathway enrichment analysis.**
