# Supplementary figures and images for: A molecular subtyping associated with the cGAS-STING pathway provides novel perspectives on the treatment of ulcerative colitis
Source: Sci Rep. 2024 Jun 3;14:12683. doi: 10.1038/s41598-024-63695-4 (PMC11148070; doi:10.1038/s41598-024-63695-4)

# B

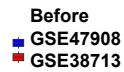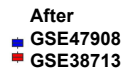

Supplement: Supplementary file 2 — Supplementary Figure S1. [file 41598_2024_63695_MOESM2_ESM.pdf]
